# SnO_2_/Graphene Nanoplatelet Nanocomposites: Solid-State Method Synthesis With High Ethanol Gas-Sensing Performance

**DOI:** 10.3389/fchem.2018.00337

**Published:** 2018-08-09

**Authors:** Run Zhang, Jian-Bo Jia, Jian-Liang Cao, Yan Wang

**Affiliations:** ^1^The Collaboration Innovation Center of Coal Safety Production of Henan Province, Henan Polytechnic University, Jiaozuo, China; ^2^Department of Chemical Engineering and Technology, College of Chemistry and Chemical Engineering, Henan Polytechnic University, Jiaozuo, China; ^3^School of Safety Science and Engineering, State Key Laboratory Cultivation Base for Gas Geology and Gas Control, Henan Polytechnic University, Jiaozuo, China

**Keywords:** Graphene nanoplatelet, SnO_2_ nanoparticles, nanocomposites, ethanol, sensitivity

## Introduction

Graphene nanoplatelet (GNP) is a well-known 2D carbon nanomaterial composed of network structure of sp^2^-hybridized carbon atoms. So far, two main strategies (exfoliation and chemical oxidation-reduction) have been used for the preparation of GNP from graphite. Unfortunately, a lot of defects are caused by the strong oxidizing reagents on the GNP produced by chemical oxidation-reduction method, and such GNP lost the remarkable electrical and mechanical properties (Coleman, 2013). Exfoliation method could be adopted to product high-quality GNP from graphite, but the yield of GNP is quite low (~1 wt.%) (Hernandez et al., 2008). Hence, the requisite large scale production of high-quality GNP remains a challenging task.

Even so, GNP is expected as a promising material for gas sensing due to its unrivaled physiochemical and electronic properties such as excellent flexibility, large specific surface area and high conductivity. Nevertheless, owing to the poor gas-sensing selectivity, GNP exhibited responses to several kinds of gas (Yoon et al., 2012; Nemade and Waghuley, 2013). But when GNP was incorporated with other sensor materials, like metal oxide semiconductors (MOSs), it could remarkably improve the sensing performance of the sensor materials (Eom et al., 2017; Thu et al., 2018). The MOSs phase facilitates the adsorption/desorption process of tested gas, thereby activating the reactions occurring on the carbon surface, which in turn increases the response speed and response/recovery time. In addition, n-p junctions can be formed by GNP with n-type metal oxides, and the resulting novel nanostructures perform much better gas sensing performance than single materials (Neri et al., 2013).

As an n-type metal oxide semiconductor, tin dioxide (SnO_2_) has a wide band gap of Eg = 3.6 eV and excellent optical and electrical properties. SnO_2_ have been one of the most extensive studied materials due to its wide applications including in transparent conductive electrodes and transistors (Liu et al., 2018; Satoh et al., 2018), lithium-ion batteries (Zhao et al., 2016; Shi et al., 2017), dye-sensitized solar cells (Hagfeldt et al., 2010), photocatalysis (Aslam et al., 2018; Praus et al., 2018) and gas sensors (Narjinary et al., 2017; Long et al., 2018; Xu et al., 2018). For gas sensing application, SnO_2_ and SnO_2_ based composites also show admirable gas sensing properties like low-cost, low detection limit, fast response and recovery, high response and good stability (Yan et al., 2015; Cao et al., 2017; Kim et al., 2017).

Herein, we put forward a simple and potentially scalable method to obtaining massive high-quality GNP from exfoliation of flake graphite in K_2_FeO_4_/H_2_SO_4_, and use the solid-state method to synthesize SnO_2_ decorated graphene nanoplatelet nanocomposites (SnO_2_/GNP) with different mass ratio of SnO_2_ and GNP. The as-prepared SnO_2_/GNP nanocomposites possess the two-dimensional (2D) structure, and the 2D GNP accelerating the preferential growth and preventing the agglomeration of the SnO_2_ nanoparticles. The gas sensing tests indicated that the sensors based on GNP/SnO_2_ nanocomposites possess high sensitivity and excellent characteristic of response and recovery toward ethanol vapor. The sensor response was found to be dependent on the mass ratio of GNP in the composites and it reaches the maximum response when the mass percentage of GNP in the composites is 5%.

## Experimental

### Preparation of the GNP/Sno_2_ nanocomposites

All the reagents were of analytical grade (AR) and used as received without further purification. The preparation of GNP was listed in the Supplementary Material. A typical synthesis process of GNP/SnO_2_ nanocomposites with 5 wt.% GNP content can be described as follows: 7 g of SnCl_4_·5H_2_O, 0.15 g of GNP and 6 ml of PEG-400 were mixed adequately and ground together in an agate mortar. Subsequently, 3.2 g of NaOH was slowly added to the mixtures and ground together for about 30 min. The reaction started readily during the addition process of NaOH, accompanied by release of heat. As the reaction proceeded, the mixture became mushy. Then samples were collected, washed several times with distilled water and absolute ethanol, and dried at 60°C overnight in a drying oven. Finally, the product was ground to powder, marked as GNP/SnO_2_-5. 2.5 and 7.5 wt.% GNP of GNP/SnO_2_ nanocomposites were prepared using the aforementioned method, and marked as GNP/SnO_2_-2.5 and GNP/SnO_2_-7.5, respectively. For comparison, the same method was used to synthesize SnO_2_ nanoparticles without GNP.

The characterization, sensor fabrication and measurement (Figure S1) were listed in the Supplementary Material.

## Results and discussion

**Figure 1:**
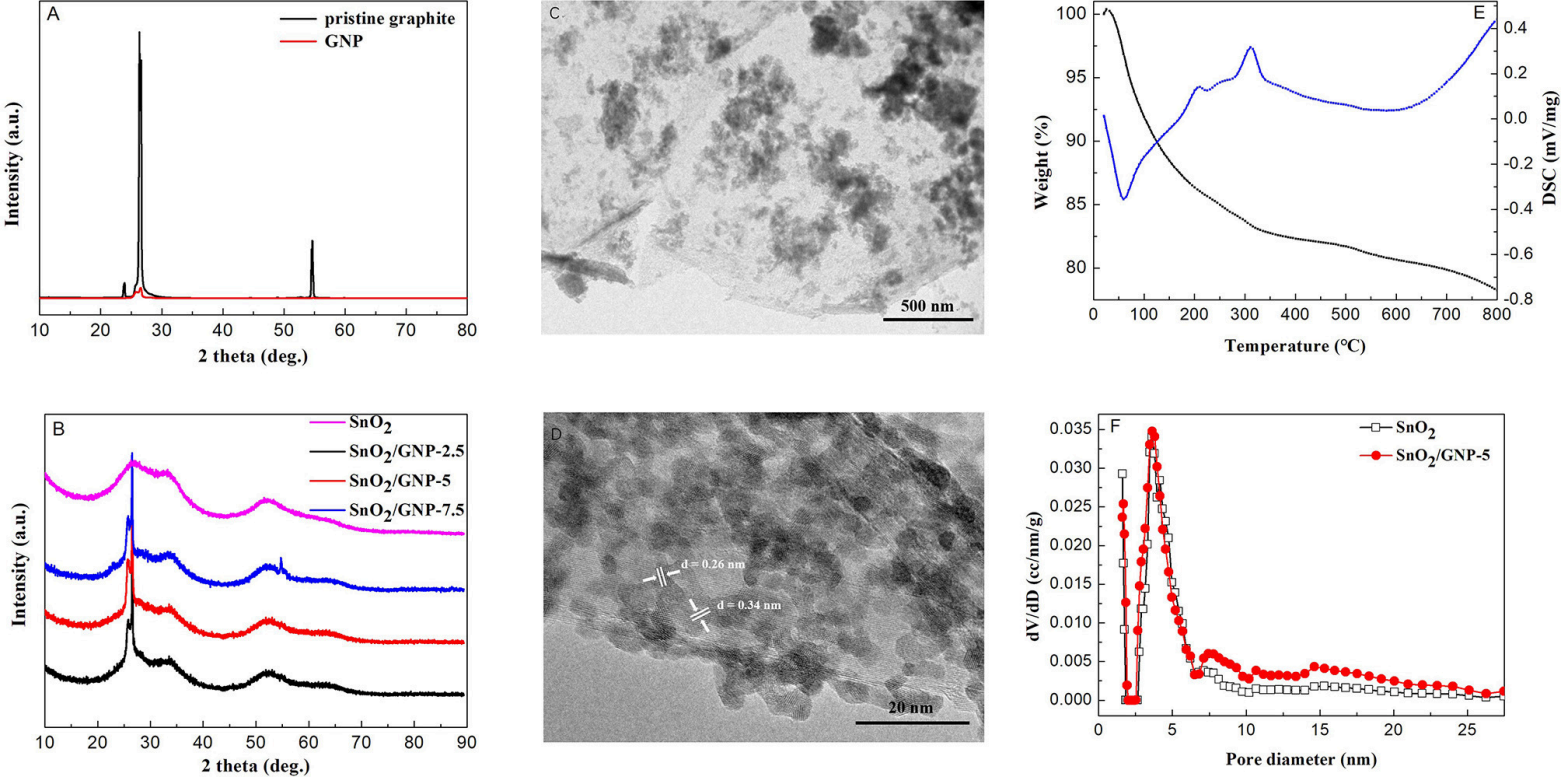
**(A)** XRD patterns of pristine graphite and GNP. **(B)** XRD patterns of SnO_2_ and the SnO_2_/GNP nanocomposites with different GNP contents. **(C,D)** TEM and HRTEM images of the SnO_2_/GNP-5 nanocomposite. **(E)** TG-DSC profiles of the SnO_2_/GNP-5 nanocomposites. **(F)** The pore size distribution curves of the SnO_2_ and SnO_2_/GNP-5 nanocomposite.

The XRD diffraction pattern of GNP is almost identical to that of pristine graphite (Figure 1A), revealing that no structural change occurred during the exfoliating process. The intensity of (002) peak centered at 26.5° of GNP decreases obviously compared with that of pristine graphite due to the ultrathin thickness of GNP (Zhang et al., 2016). The XRD diffraction patterns of pure SnO_2_ nanoparticles and GNP/SnO_2_ nanocomposites are shown in Figure 1B. We can see that four distinct diffraction peaks of SnO_2_ centered at 2θ of 26.6°, 33.9°, 51.7°, and 65.9°, which are corresponding to the reflection from the (110), (101), (211), and (301) planes of the tetragonal rutile SnO_2_ (JCPDS Card No. 41-1445), respectively. This confirmed that the synthesis method that SnO_2_ was successfully prepared by solid-state reaction is feasible and complete. However, as seen from Figure 1B, there are no diffraction peak around 26.6° of SnO_2_ observed in the curves, because the diffraction peaks of 26.5° of GNP is so high that the peak around 26.6° of SnO_2_ is covered.

Figure S2a shows the representative FESEM image of pristine graphite. In Figure S2b (FESEM image of GNP), two-dimensional (2D) structure of the thin layers can be seen clearly. As shown in Figure S2c, the FESEM image of the pure SnO_2_ exhibits particles with the size of 100–200 nm. The FESEM and TEM images of the GNP/SnO_2_-5 nanocomposite are presented in Figure S2d and Figure 1C, respectively, and which show that numerous particles are dispersed on the surface of 2D sheets of GNP. Meanwhile, as can be seen from Figure 1D, two phases of GNP and SnO_2_ are clearly observed and closely in contact to form an intimate interface. And, the lattice fringes with interplanar spacings of 0.26 nm and 0.34 nm can be corresponding to the (101) and (110) planes of SnO_2_ nanoparticles. It can be concluded that the GNP/SnO_2_ composites were synthesized successfully using the solid-state method.

TG-DSC analysis revealed the weight change situation of GNP/SnO_2_-5 nanocomposites from room temperature to 800°C with the heating rate of 10°·min^−1^. As is shown in Figure 1E, there are two stages of weight loss in the TG curve according to the peaks of DSC curve. The first stage in temperature before 300°C is due to desorption of moisture and solvent. The second stage of weight loss is due to the combustion of GNP in air. This result proves that the GNP/SnO_2_-5 nanocomposite was not decomposed at the operating temperature of 280°C in the procedure of measuring gas-sensing properties.

Figure 1F displays the pore diameter distribution of the SnO_2_ and GNP/SnO_2_-5 samples. It can be clearly seen that the pore diameters of pure SnO_2_ and GNP/SnO_2_-5 are relatively small, which both the majority concentrate on about 2 nm and 4 nm. The specific surface areas of GNP/SnO_2_-5 sample is 167.01 m^2^·g^−1^, which is higher than SnO_2_ (119.67 m^2^·g^−1^). Increasing specific surface area could be in favor of enhancing gas-sensing properties.

**Figure 2:**
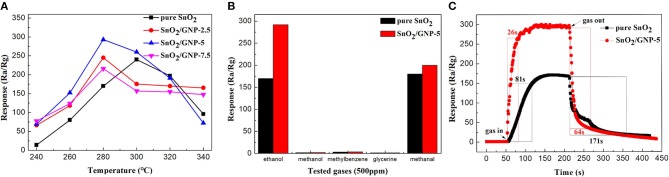
**(A)** The response values of SnO_2_, SnO_2_/GNP-2.5, SnO_2_/GNP-5, and SnO_2_/GNP-7.5 sensors toward 500 ppm of ethanol at different working temperatures. **(B)** The responses of sensors (SnO_2_ and SnO_2_/GNP-5) operated at 280°C in 500 ppm of different gases. **(C)** The response-recovery curve of SnO_2_ and SnO_2_/GNP-5 toward 500 ppm of ethanol at 280°C.

Figure 2A shows the response values of pure SnO_2_ nanoparticles-based sensor and GNP/SnO_2_-based sensors to 500 ppm of ethanol at different temperatures. From the curves of GNP/SnO_2_-2.5, GNP/SnO_2_-5, and GNP/SnO_2_-7.5, it can be clearly observed that the response values increased with the increase of the temperature. However, the response values decrease when the temperature is above 280°C. As a result, the best operating temperature of GNP/SnO_2_-based sensors is 280°C. Similarly, the best operating temperature of pure SnO_2_ sensors is 300°C. We can get a conclusion that the best operating temperature is lowered 20°C because of the joining of GNP. Compared between the different curves, it reaches the maximum response when the mass percentage of GNP in the composites is 5%. The response value of GNP/SnO_2_-7.5 sample is lower than that of the GNP/SnO_2_-5 sample. It is because that activation center still focuses on the SnO_2_ nanoparticle, and the high content of GNP may lead to the decrease of SnO_2_ nanoparticle on the unit specific surface area. Some SnO_2_-based materials of ethanol sensing from the literature are summarized in Table S1. It can be observed that the GNP/SnO_2_ composite exhibits superior performances compared with other SnO_2_-based materials.

Figure S3 displays the response values of sensors based on pure SnO_2_ and GNP/SnO_2_-5 to different concentrations of ethanol at 280°C. As shown in the curves, the response values of the two sensors increased with the increasing of ethanol concentrations in the range of 50–2,000 ppm. We can find its regularity through a large number of relevant experiments to establish the relationship between response value and concentration of ethanol. From comparison of two curves, a gradual enhancement in response amplitude was observed for both sensors, and the response amplitudes of GNP/SnO_2_-5 based sensor are always higher than that of pure SnO_2_, demonstrating its better sensitivity to ethanol.

It is well known that selectivity is another key criterion for measuring the quality of gas sensors. Figure 2B shows the selectivity test results of the pure SnO_2_ and GNP/SnO_2_-5 sensors to five different gases of 500 ppm, including methanol, ethanol, methylbenzene, glycerine and methanal. It can be observed that the GNP/SnO_2_-5-based sensor has good selectivity to ethanol compared to that of pure SnO_2_ sensor at 280°C. The higher response to ethanol may be because ethanol is more likely to lose electrons in the process of a redox reaction with the absorbed oxygen, and the hydroxyl group (–OH) is much easier to oxidize at the optimum operating temperature.

The response–recovery time curve of GNP/SnO_2_-5-based sensor to 500 ppm of ethanol is shown in Figure 2C. Response and recovery time are defined as change in the resistances from Ra to [Ra−90% × (Ra – Rg)] for gas-in and [Ra + 90% × (Ra – Rg)] to gas-out, respectively (Zhang S.S. et al., 2018; Zhang Y.J. et al., 2018). It can be clearly observed that the response increased and decreased quickly when the GNP/SnO_2_-5-based sensor was exposed to and separated from ethanol, respectively. The response time and the recovery time of GNP/SnO_2_-5-based sensor are 26 and 64 s, respectively, which are much shorter than of the pure SnO_2_-based sensor that are 81 and 171 s. The relatively rapid response and recovery time could be due to the unique structure, which is the SnO_2_ nanoparticles are decorated on the 2D sheet of GNP. This indicates that the large specific surface area is favorable to the adsorption of ethanol, which verifies the above conjecture. Figure S4 depicts the response values of GNP/SnO_2_-5-based sensor to 500 ppm of ethanol for every 3 days in 30 days at 280°C, which fall slightly but are maintained around 295. Therefore, the conclusion could be obtained that the GNP/SnO_2_-5-based gas sensor to ethanol has a satisfactory stability, which confirms that the sensor might have a practical application.

## Conclusion

In conclusion, we reported an easy method to successfully prepare massive high-quality GNP from exfoliation of flake graphite, and GNP/SnO_2_ nanocomposites were successfully synthesized by a facile solid-state method. The 2D GNP has no structural change during the exfoliating process from flake graphite, and the SnO_2_ nanoparticles were highly distributed on the surface of GNP. The GNP/SnO_2_ based sensor showed excellent gas sensing performance toward ethanol, and the ameliorative gas-sensing properties may be due to the accrescent specific surface area and the interaction between 2D GNP and SnO_2_ nanoparticles. Due to the procedure is convenient and environment-friendly, and good gas sensing property of the SnO_2_/GNP nanocomposite, it could be a promising candidate for ethanol detection.

## Author contributions

RZ performed the experiments and analyzed the data with the help from J-LC. J-BJ and YW conceived the study. All authors discussed the results and wrote the manuscript.

### Conflict of interest statement

The authors declare that the research was conducted in the absence of any commercial or financial relationships that could be construed as a potential conflict of interest.
