# Supplementary material for: SnO2/Graphene Nanoplatelet Nanocomposites: Solid-State Method Synthesis With High Ethanol Gas-Sensing Performance
Source: Front Chem. 2018 Aug 9;6:337. doi: 10.3389/fchem.2018.00337 (PMC6095040; doi:10.3389/fchem.2018.00337)
Supplement: Supplementary file 1 [file Presentation_1.PDF]

Supplementary Material

SnO_2/Graphene Nanoplatelet Nanocomposites: Solid-State Method Synthesis with High Ethanol Gas-Sensing Performance

Run Zhang[1,2], Jian-Bo Jia[1,2*], Jian-Liang Cao[1,2] and Yan Wang[1,3*]

[1] The Collaboration Innovation Center of Coal Safety Production of Henan Province, Henan Polytechnic University, Jiaozuo, China, [2] College of Chemistry and Chemical Engineering, Henan Polytechnic University, Jiaozuo, China, [3] School of Safety Science and Engineering, State Key Laboratory Cultivation Base for Gas Geology and Gas Control, Henan Polytechnic University, Jiaozuo, China

***Correspondence: jiajianbo@hpu.edu.cn (J. J.); yanwang@hpu.edu.cn (Y.W.)**

Experimental

Materials

K_2FeO_4 was purchased from Macklin Biochemical Co., Ltd. (Shanghai, China). Graphite flake with an average size of 100 mesh was supplied by Qingdao graphite company (Shandong, China). Tin(IV) chloride pentahydrate ($SnCl_4·5H_2O$, 99.0%), sodium hydroxide (NaOH), and polyethylene glycol 400 (PEG-400) were purchased from Sinopharm Chemical Reagent Co., Ltd (Beijing, China).

Preparation of GNP

K_2FeO_4, a hexavalent ironsalt, is a powerful oxidizing agent, and its oxidizing ability is strongly dependent on pH, especially in an acidic environment, where its redox potential is the highest (Mao et al., 2006). K_2FeO_4/H_2SO_4 is an effective, nondestructive and green oxidation system for oxidative functionalization of carbon nanotubes (Zhang et al., 2015). In this oxidation system, the sp^3-hybridized carbon atoms were selectively oxidized, but the C=C bonds were entirely unaffected. Although sp^2-hybridized carbon atoms wouldn't be oxidized in K_2FeO_4/H_2SO_4, FeO_4^{2-} was formed whose penetrability was strong and could speedily intercalate into the interlayer spacing of graphite (Peng et al., 2015). Furthermore, $FeO4^{2-}$ reacts with H_2O to produce O_2, and the reaction rate is high. The instantaneously excessive

pressure of O_2 in the interlayer spacing of graphite might weaken the conjugate forces between pristine graphitic lamellae. So herein, we propose a simple and fast methodology for the preparation of GNPs via K_2FeO_4 as an intercalator.

GNP was prepared by following steps: 1.0 g flake graphite and 30.0 mL of concentrated sulfuric acid were loaded into a 50 mL beaker, then 5.0 g K_2FeO_4 was slowly added to the mixture at room temperature under magnetic stirring. The mixture was sonicated for 1h and stirred at 40 ^{O}C for 3h. The resulting mixture was centrifuged, the precipitate was poured into 200 mL of cold water, and settled for 2 h to sediment the solid GNP. After repeated centrifugation and water-washing, the obtained GNP was freeze-dried, collected and stored in a desiccators.

Characterization

The phase and crystallinity of the samples were examined using X-ray diffraction (XRD, Cu Kα, Bruker-AXSD8) (Bruker, Madison, WI, USA) with a wavelength of 0.154 nm in step of 10°(2θ) min^{-1} from 10° to 90°(2θ). The morphologies and structures of the as-prepared products were directly observed through field emission scanning electron microscopy (FESEM, Quanta™250 FEG) (FEI, Eindhoven, The Netherlands) and transmission electron microscopy (TEM, JEOL JEM-2100 microscope) (JEOL, Tokyo, Japan). Thermal gravity and differential scanning calorimeter (TG–DSC) was carried out on a TA-SDT Q600 (TA Instruments, New Castle, DE, USA) at a heating rate of 10 °Cmin^{-1}at an air atmosphere. The porous feature of the samples were characterized by the N_2 adsorption-desorption measurement (Quantachrome Autosorb-iQ sorption analyzer) (Quantachrome, Boynton Beach, FL, USA). Before carrying out the measurement, the samples were degassed at 100°C for more than 12 h. The specific surface areas (S_{BET}) and the pore diameter distributions were determined from Nitrogen adsorption–desorption isotherms.

Sensor fabrication and measurement

The gas sensing property of the as-prepared composite to ethanol was tested by the intelligent gas-sensing analysis system (CGS-4TPs) (Beijing Elite Co., Ltd., Beijing, China). Figure S1 shows a simple device schematic diagram. The relative humidity is 34% and the temperature is 25 °C in the test chamber in the process of the gas-sensing test. The gas sensors were made in a conventional way (Sun et al., 2016). The way is

demonstrated as follows: a small amount of the as-prepared products were fully ground in an agate mortar with a few drops of ethanol. Then, the pastes were equably spread on a ceramic substrate (13.4 mm × 7 mm) with interdigitated Ag-Pd electrodes to form the thin film. To improve the stability and repeatability of the gas sensors, the substrate was aged at 180 °C for 24 h before starting the test. Response of the gas sensor was defined as: Response = Ra/Rg (Where, Ra and Rg were the resistances of the sensor measured in air and in test gas, respectively.)

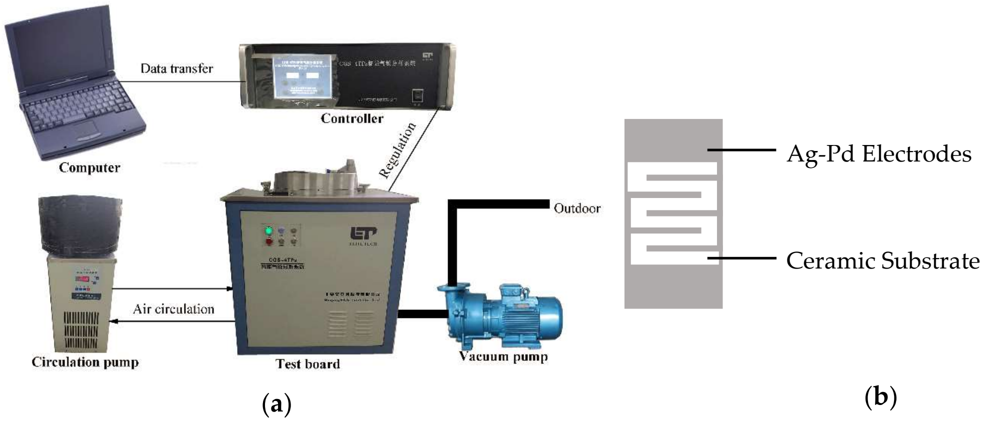

FIGURE S1 (a) The CGS-4TPS gas-sensing test system, and (b) the gas sensor substrate.

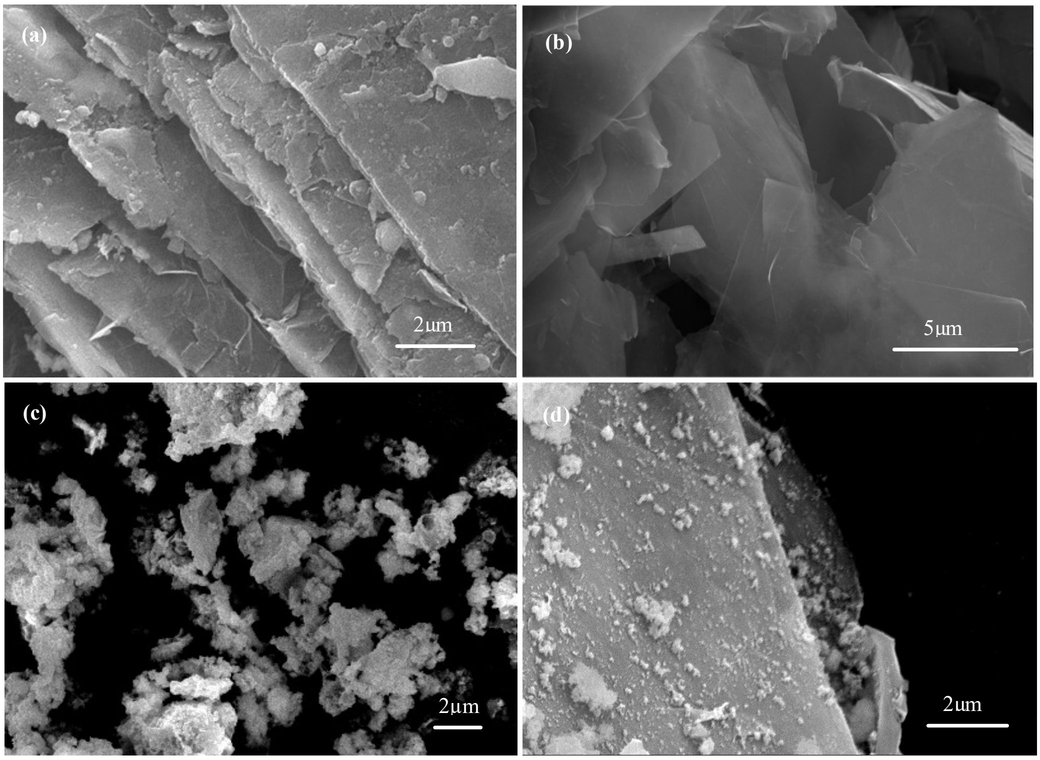

FIGURE S2 Scanning electron microscope (SEM) images of (a) pristine graphite, (b) GNP, (c) SnO_2 nanoparticles, and (d) SnO_2/GNP-5 nanocomposite.

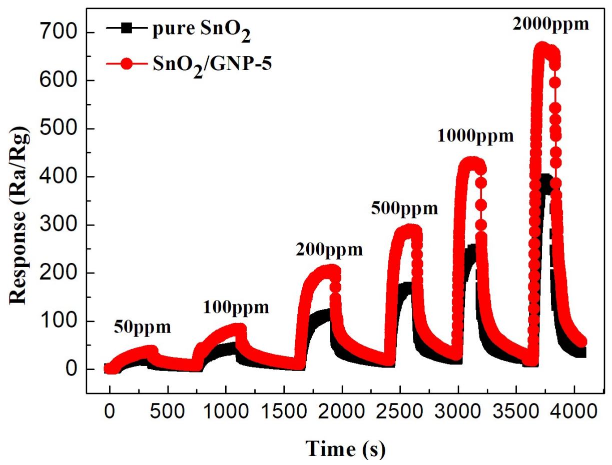

FIGURE S3 The responses of sensors (SnO_2 and SnO_2/GNP-5) versus different concentrations of ethanol at 280 °C.

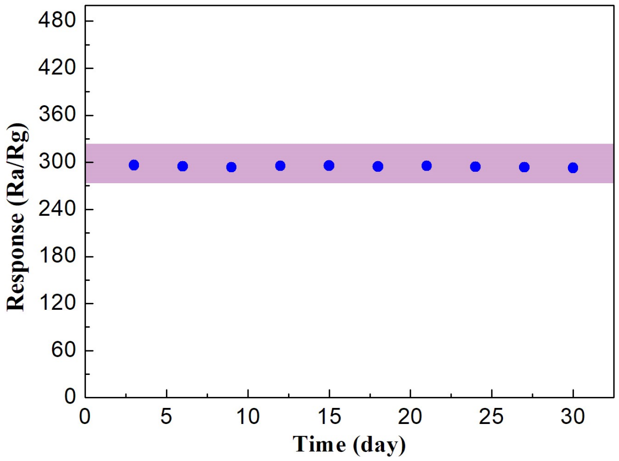

FIGURE S4 The stability curve of SnO_2/GNP-5 to 500 ppm of ethanol in 30 days.

TABLE S1 Comparison of the gas sensing properties of various SnO_2-based material to ethanol.

Sensing materials	Ethanol concentration (ppm)	Temperature (°C)	Response (Ra/Rg)	Ref.
SnO_2 nanowires	100	360	31	[33] (Phadungdhitidhada et al., 2011)
SnO_2 nanotubes	200	300	16.7	[34] (Zhang et al., 2013)
SnO_2 nanorods	100	300	30.7	[35] (Chen et al., 2006)
SnO_2 nanoflowers	100	300	47.29	[36] (Yu et al., 2016)
SnO_2 nanoflowers	200	240	62.2	[37] (Li et al., 2016)
RGO/hollow SnO_2	100	300	70.4	[38] (Zito et al., 2017)
Pure SnO_2	100	280	46	this work
SnO_2/GNP-5	100	280	94.6	this work

References

Mao, W., Wang, J., Xu, Z., Niu, Z., Zhang, J. (2006). Effects of the oxidation treatment with K_2FeO_4 on the physical properties and electrochemical performance of a natural graphite as electrode material for lithium ion batteries. *Electrochem. Commun.* 8, 1326–1330. doi: 10.1016/j.elecom.2006.06.011

Zhang, Z., Xu, X. (2006). Nondestructive covalent functionalization of carbon nanotubes by selective oxidation of the original defects with K_2FeO_4. *Appl. Surf. Sci.* 346, 520–527. doi: 10.1016/j.apsusc.2015.04.026

Peng, L., Xu, Z., Liu, Z., Wei, Y., Sun, H., Li, Z., et al. (2015). An iron-based green approach to 1-h production of single-layer graphene oxide. *Nat. Commun.* 6, 5716. doi: 10.1038/ncomms6716

Sun, G., Chen, H., Li, Y., Ma, G., Zhang, S., Jia, T., et al. (2016). Synthesis and triethylamine sensing properties of mesoporous α-Fe_2O_3 microrods. *Mater Lett.* 178, 213–216. doi: 10.1016/j.matlet.2016.04.209

Phadungdhitidhada, S., Thanasanvorakun, S., Mangkorntong, P., Choopun, S., Mangkorntong, N., Wongratanaphisan, D. (2011). SnO_2 nanowires mixed nanodendrites for high ethanol sensor response. *Curr. Appl. Phys.* 11, 1368–1373. doi: 10.1016/j.cap.2011.04.007

Zhang, J., Guo, J., Xu, H., Cao, B. (2013). Reactive-template fabrication of porous SnO_2 nanotubes and their remarkable gas-sensing performance. *ACS Appl. Mater. Interfaces* 5, 7893–7898. doi: 10.1021/am4019884

Chen, Y., Nie, L., Xue, X., Wang, Y., Wang, T. (2006). Linear ethanol sensing of SnO_2 nanorods with extremely high sensitivity. *Appl. Phys. Lett.* 88, 083105. doi: 10.1063/1.2166695

Yu, X., Zeng, W. (2016). Fabrication and gas-sensing performance of nanorod-assembled SnO_2 nanostructures. *J. Mater. Sci.: Mater. Electron.* 27, 7448–7453. doi: 10.1007/s10854-016-4721-0

Li, T., Zeng, W., Long, H., Wang, Z. (2016). Nanosheet-assembled hierarchical SnO_2 nanostructures for efficient gas-sensing applications. *Sens. Actuat. B* 231, 120–128. doi: 10.1016/j.snb.2016.03.003

Zito, C.A., Perfecto, T.M., Volanti, D.P. (2017). Impact of reduced graphene oxide on the ethanol sensing performance of hollow SnO_2 nanoparticles under humid atmosphere. *Sens. Actuat. B* 244, 466–474. doi: 10.1016/j.snb.2017.01.015
